# Liver transplantation in glycogen storage disease type I

**DOI:** 10.1186/1750-1172-9-47

**Published:** 2014-04-09

**Authors:** Susanna JB Boers, Gepke Visser, Peter GPA Smit, Sabine A Fuchs

**Affiliations:** 1 Department of Metabolic Diseases, Wilhelmina Children’s Hospital, University Medical Center Utrecht, Lundlaan 6, 3584 EA Utrecht, The Netherlands; 2 Beatrix Children’s Hospital, University Medical Centre Groningen, Hanzeplein1, 9700 Groningen, The Netherlands

## Abstract

Glycogen storage disease type I (GSDI), an inborn error of carbohydrate metabolism, is caused by defects in the glucose-6-transporter/glucose-6-phosphatase complex, which is essential in glucose homeostasis. Two types exist, GSDIa and GSDIb, each caused by different defects in the complex. GSDIa is characterized by fasting intolerance and subsequent metabolic derangements. In addition to these clinical manifestations, patients with GSDIb suffer from neutropenia with neutrophil dysfunction and inflammatory bowel disease.

With the feasibility of novel cell-based therapies, including hepatocyte transplantations and liver stem cell transplantations, it is essential to consider long term outcomes of liver replacement therapy. We reviewed all GSDI patients with liver transplantation identified in literature and through personal communication with treating physicians. Our review shows that all 80 GSDI patients showed improved metabolic control and normal fasting tolerance after liver transplantation. Although some complications might be caused by disease progression, most complications seemed related to the liver transplantation procedure and subsequent immune suppression. These results highlight the potential of other therapeutic strategies, like cell-based therapies for liver replacement, which are expected to normalize liver function with a lower risk of complications of the procedure and immune suppression.

## Introduction

Glycogen storage disease type I (GSDI) is an autosomal recessive inborn error of carbohydrate metabolism caused by defects in the glucose-6-phosphate transporter (G6PT)/glucose-6-phosphatase (G6Pase) complex [1,2]. G6PT/G6Pase complex plays a crucial role in interprandial glucose homeostasis and consists of a catalytic subunit, glucose-6-phosphatase-α (G6Pase-α) encoded by the *G6PC* gene and a glucose-6-phosphatase transporter (G6PT), encoded by the *SLC37A4* gene. Deficient activity of G6Pase-α causes GSDIa [3] and deficient activity of G6PT causes GSDIb [4]. GSDI is a relatively rare disorder with an incidence of 1:100.000, represented in 80% of the patients by GSDIa and in 20% by GSDIb [5].

G6Pase catalyzes the final step in glycogenolysis and in gluconeogenesis in the lumen of the endoplasmic reticulum in primarily liver, but also kidney and intestine, by hydrolyzation of glucose-6-phosphate (G6P) to glucose and inorganic phosphate. Because G6Pase affects both glycogenolysis and gluconeogenesis, inactivating mutations in the *G6PC* or the *SLC37A4* gene result in severely reduced fasting tolerance. Clinical complications in patients include hepatomegaly, nephromegaly, hypoglycemia, hyperlipidemia, hyperuricemia, lactic acidemia, and growth retardation [5]. In addition to the clinical manifestations in GSDIa, patients with GSDIb generally also suffer from neutropenia, impaired neutrophil function and inflammatory bowel disease.

Prevention of hypoglycemia is crucial in the treatment of GSDI [5]. This is achieved by frequent feedings during day and night or nocturnal gastric drip feeding. Any feeding problem can result in a hypoglycemic event, with risk of cognitive impairment, seizures and finally death. This represents a constant threat for patients and their parents, severely affecting quality of life.

Despite progress in the treatment of GSDI, metabolic control remains challenging and hepatic, renal and/or immunologic complications may arise. Because of the prominent hepatic manifestations in GSDI, orthotopic liver transplantations have been performed [5]. Short-term outcome of liver transplantation for GSDI is encouraging, but very few papers report long-term follow-up. With the advent of less invasive cell-based therapies, including hepatocyte transplantations and liver stem cell transplantations, which might become a therapeutic option for all patients with GSDI, it is eminent to know the long-term outcomes of liver specific therapies. We therefore reviewed short-term and long-term outcomes of liver transplantations in GSDIa and GSDIb patients.

## Methodology

English-language literature was systematically reviewed through searches in PubMed and in the references of relevant publications to find all GSDIa and GSDIb liver transplantations published in literature. Through personal communication with treating physicians, we completed information and identified additional cases.

## Results

We identified 58 patients with GSDIa who underwent a liver transplantation between 1982 and 2012 (Table 1, Additional file 1: Table S1); 3 of these patients received a second liver transplantation. The average age at transplantation was 20 years (range: 4.3-50 years). A living-related transplantation was performed in 16 cases. 6 Patients underwent a combined liver-kidney transplantation (Table 2). The immunosuppressive regime consisted of steroids, combined with cyclosporine in 12 patients, with cyclosporine and azathioprine in 17 patients and with tacrolimus in 9 patients. The specific immunosuppressant medication was not reported in 22 cases (Table 1).

**Table 1:** Indication and follow-up of GSDIa and GSDIb liver transplantation

	**GSDIa (n = 58)**	**GSDIb (n = 22)**
**Year of transplantation**	**1982 – 2012**	**1991 – 2012**
**Indications**		
Hepatic adenomas or liver abnormalities (mostly focal nodular hyperplasia)	29 [6-22]	
Poor metabolic control	27 [6,8-10,14-16,19,21,23-27]	21 [13,28-34]
Growth retardation	13 [10,14-16,23,24,27]	3 [32,33,35]
Recurrent infections		10 [28,33,34]
Renal failure	5 [36]	
-Of which also kidney transplant	3 [17,26,37]	
Bleeding complications (anemia)	1 [27]	
Pancreatitis	1 [24]	
Anal and oral ulcera		1 [30]
**Immunosuppressive regime**		
Cyclosporine	12 [6,7,9,10,12,14,15,17],[21,26]	1 [28]
Cyclosporine + azathioprine	17 [16,23,25,38,39]	-
Tacrolimus	9 [16,18,21,36]	16 [30,31,33-35]
Not reported	22	6
**Outcome and complications GSDIa**	**Short-term (≤1 year)**	**Long-term (>1 year)**
Normalization liver function	58 (all)	-
Catch-up growth	-	13 [10,13,14,23,25,39]
Sexual maturation	-	2 [25]
Re-OLT	2 [16,23]	1 [20]
Death	1 (pancreatitis and sepsis) [25]	3 (rejection related liver failure, suicide, metastatic hepatocellular carcinoma) [19], ^PC^
Renal failure	6 [21]	4 [20]
-Of which already pre-transplant	1 [39]	
-Requiring dialysis	1 [12] (temporary)	2 [27], ^PC^ (permanent)
-Gouty arthritis (due to renal failure?)	-	1 [14]
Metastatic hepatocellular carcinoma	1^PC^	
Transplantation associated		
-Hepatic artery thrombosis	2 [16,23]	-
-Portal vein thrombosis	-	2 [6,16]
-Hepatic vein obstruction	2 [25]	-
-Prolonged drainage	2 [25,39]	-
-Rejection	5 [14,20,21,38,39]	3 [6,9]
-Never functioning liver after transplantation	2 [23,24]	
Therapy (immune suppression) associated		
-Hypertension	4 [25,39]	-
-Insulin-dependent diabetes (reversible)	3 [8,9,21]	-
-Infections (CMV, hepatitis)	7 [21,25,39]	1 [14]
**Outcome and complications GSDIb**	**Short-term (≤1 year)**	**Long-term (>1 year)**
Normalization liver function	23 (all)	
Catch-up growth		2 [32]
Death	1 (systemic candidiasis) [40]	
Neutropenia	-	
-Improved	-	14 [31,34,35]
-Persisted		7 [13,29,30,32,33]
Thrombocytopenia		1 [28]
Transplantation associated		
-Infection	6 [34]	-
-Anemia	1 [33]	-
-Rejection	-	1 [13]
Therapy (immune suppression) associated		
-Infection	1 [30]	1 [26]
-Tacrolimus encephalopathy	1 [35]	

**Table 2:** Follow-up of combined liver-kidney transplantation

**Combined liver-kidney transplantation**	**Year of transplantation [ref]**	**Age at transplantation**	**Current age (if alive)**	**Outcome and complications**
**GSDIa**	1996 (2nd OLT and KT) [20]	30	†	1.3 years: PT died at age 31
	1996 [37]	34	52	4.1 years: alive
	2000 [26]	19.5	34	2 years: normal liver and kidney function
	2004 (publication) [17]	25	35	4 months: normal liver and kidney function
	2011 (publication) [36]	30	33	7 months: good condition, both grafts functional
**GSDIb**	2003 (2y later OLT) [33]	32	41	8 months: good liver function and normal kidney function

The indication for liver transplantation varied and included hepatic adenomas/liver abnormalities/focal nodular hyperplasia (29 patients), poor metabolic control (27 patients), growth retardation (13 patients, some with delayed puberty and sexual maturation), renal failure (5 patients, 3 of whom received a combined liver-kidney transplantation), bleeding complications leading to anemia (1 patient) and acute pancreatitis due to severe hypertriglyceridemia (1 patient).

4 Patients with GSDIa died; 1 due to rejection related liver failure (15 years after 1^st^ liver transplantation, 1 year after 2^nd^ liver transplantation with combined kidney transplantation), 1 committed suicide 3 years post-transplantation, 1 because of metastatic hepatocellular carcinoma 4 months after transplantation, and 1 because of pancreatitis and sepsis 2 months post-transplantation. All other patients were alive at time of follow-up (range several months to 11.3 years post-transplantation). In all cases, liver function was good and metabolic control normalized, without a specific dietary regime. In 13 cases, catch-up growth was mentioned (Table 3). The patients with catch-up growth reported were all children or teenagers and for 2 of these, sexual maturation was also reported. In addition, one patient of 27 years old showed an increase of 5.4 cm in height 2 years after transplantation.

**Table 3:** Follow-up of catch-up growth

**Catch-up growth**	**Year of transplantation [ref]**	**Age at transplantation**	**Current age (if alive)**	**Catch-up growth**
**GSDIa**	1986 [23]	6	34	2 years: catch-up growth
	1987 [10]	27	54	2 years: catch-up growth (5.4 cm)
	1993 [13]	11.8	33	Yes: time of follow-up not mentioned
	Between June 1994 and December 2005 [25]	median 7.3 (n = 4)	Unknown	The mean height-for-age increased from <10^th^ percentile (at t = 0) to 50^th^ percentile at 5 years
	Between 1996 and 2001 [39]	4.3-14.5 (n = 4)	20-30	2 years: catch-up growth
	1999 (publication) [14]	15	30	8 years: catch-up length growth (-6SD to -1.5SD)
	1999 (publication) [14]	17	32	6 years: catch-up length growth (-2.5SD to -1.5SD)
**GSDIb**	2004 (publication) [32]	8	18	Yes: time of follow-up not mentioned
	2004 (publication) [32]	11.1	21	Yes: time of follow-up not mentioned

The complication reported most frequently was acute or chronic renal failure. Acute renal failure occurred in 8 patients, including 1 patient with pre-transplantation renal failure. One of these 8 patients required temporary dialysis. Chronic renal failure was seen in 6 patients after transplantation and 2 of these patients required dialysis. In addition, 1 case of gouty arthritis was reported 4 years post-transplantation. It is unclear whether this was due to renal failure. None of the patients who had received a combined liver-kidney transplantation developed renal failure.

Transplantation associated complications were seen in 18/58 patients and included hepatic artery thrombosis (2 patients), late portal vein thrombosis (2 patients), hepatic vein obstruction (2 patients), prolonged drainage (2 patients), acute (steroid responsive) rejection (5 patients), chronic rejection (3 patients) and a never functioning liver transplant (2 patients). Complications that might have been caused by immune suppression included hypertension (4 patients), starting one month after transplantation and reversible insulin-dependent diabetes (3 patients), starting within the first week after transplantation (Table 4). In addition, various infections were reported (5 with cytomegalovirus 1 month after transplantation and 1 patient with hepatitis 4 years after transplantation).

**Table 4:** Follow-up of therapy-associated complications

**Therapy-associated complications**	**Therapy-associated complication**	**Follow-up time [ref]**
**GSDIa**	Diabetes (n = 3)	-Acute: diabetes mellitus (1 patient) [9,21]
		-2 days: insulin-dependent diabetes (1 patient) [9]
		-3 days: for following 5 days insulin pump (1 patient) [8]
	Hypertension (n = 4)	-1 month: 4 patients, 2 received short term treatment with antihypertensive medication [25,39]
	Infection (n = 8)	-Acute: CMV (2 patients) [21]
		-1 month: CMV (5 patients) [25,36,39]
		-4 years: hepatitis C (1 patient) [14]
**GSDIb**	Infection (n = 32)	-7 months: hepatitis B (1 patients) [30]
		-6.2 years: CMV (1 patient) [20]
	Encephalitis	-Acute: tacrolimus encephalitis (1 patient) [35]

Liver transplantation was performed in 22 patients with GSDIb between 1991 and 2012, at an average age of 10 years (range 1–44 years) (Table 1, Additional file 1: Table S2). One patient had a kidney transplantation 2 years prior to the liver transplantation, with good function of both grafts reported 8 months after liver transplantation (Table 2). Indications for liver transplantation in GSDIb patients included poor metabolic control (21 patients) and/or recurrent infections (10 patients), growth retardation (3 patients), and oral and anal ulcera (1 patient). Immune suppression after transplantation involved cyclosporine in 1 patient and tacrolimus in 16 patients. The immune suppressive medication wat not reported in the other 6 patients.

At follow-up, 1 patient had died 1.4 months after transplantation, due to systemic candidiasis. In all patients, metabolic abnormalities were corrected by transplantation. Catch-up growth was reported in 2 cases (Table 3). In 14 patients, neutropenia improved, while in 7 patients neutropenia persisted. One report mentioned prolonged bleeding time and bruises after several years. 7 Patients had transplantation associated complications, including anemia in the first days after the transplantation (1 patient) and various infectious diseases shortly after transplantation (6 patients). Complications potentially associated with immune suppressive therapy included 1 patient with hepatitis B, seven months after transplantation, and 1 patient with cytomegalovirus infection several years after transplantation (Table 4). 1 patient developed tacrolimus encephalopathy soon after transplantation, which was resolved upon withdrawal of tacrolimus. None of the reports described renal complications.

## Discussion

In this review, we show that liver transplantations sustainably corrected fasting tolerance and the induced metabolic abnormalities associated with GSDIa and GSDIb, thereby immensely improving quality of life for patients. In addition, catch-up growth was seen in most patients with growth retardation (13/13 (100%) and 2/3 (67%) in GSDIa and GSDIb patients, respectively).

The extra-hepatic symptoms of the disease might however persist after liver transplantation. In GSDIa patients, renal failure was the most common complication (14/58 (24%) of patients) and 3/14 (21%) required dialysis. The natural course of renal function in GSDI patients shows a biphasic pattern [41]. We identified only 1 patient with both pre- and post-transplantation renal failure; renal function was restored in the other 4 patients with pre-transplantation renal dysfunction. Notably, 3 of these 4 patients had received a combined liver-kidney transplantation. In the other patients, renal dysfunction developed after transplantation. It is yet unclear whether post-transplantation renal failure in GSDIa represents progression of the disease, a secondary reaction to poor metabolic control, toxicity from immune suppressive medication after liver transplantation, or a combination. Strikingly, none of the GSDIb patients developed renal failure. We are unaware of a pathophysiological mechanism to explain why GSDIa patients would be more prone to develop renal failure than GSDIb patients, nor has this been observed in our experience with GSDI patients. Potentially, this is a coincidental finding due to the relatively small number of patients evaluated.

In GSDIb patients, persistent neutropenia was the most important complication (7/22 (32%) of patients). Neutropenia in GSDIb has recently been attributed to a second G6P hydrolase, called G6Pase-β [42]. The G6PT/G6Pase-β complex maintains glucose homeostasis and function in neutrophils. Deficiency of the G6PT/G6Pase-β complex in neutrophils leads to impaired endogenous glucose production and enhanced endoplasmic reticulum stress, oxidative stress and apoptosis, leading to neutropenia [42]. Migration of neutrophils from the blood to inflamed tissues (e.g. intestines, liver adenoma) might further contribute to the neutropenia [43]. It remains unclear why neutropenia improves after liver replacement in some patients and persists in others. It is possible that improved metabolic control and general well-being result in decreased inflammation, leading to higher blood neutrophil concentrations. Second, immediate increase in neutrophil count after liver transplantation might be related to the neutrophilic effect of steroid therapy. However, recurrence of neutropenia after steroid tapering has not been reported. Finally, host/donor bone marrow chimerism has been observed after liver transplantation [44]. Donor-derived leukocytes, co-transplanted with the liver graft, can migrate into the recipient’s immune system and bone marrow. This phenomenon may result in both long-term tolerance induction and induction of enzymatic activity in extra-hepatic tissue. Clearly, although the molecular mechanism causing congenital neutropenia in GSDIb has now been elucidated, many aspects of the phenotype remain poorly understood.

Our review shows that there are still many complications related to the liver transplantation procedure (18/58 (31%) in GSDIa and 8/22 (36%) in GSDIb patients), as well as complications suspected to be related to immune suppressive therapy (13/58 (22%) in GSDIa and 3/22 (14%) in GSDIb patients). In the light of these complications, novel therapeutic strategies, like hepatocyte and liver stem cell transplantations might represent attractive alternatives for liver transplantation in GSDI patients. Liver (stem) cells can be infused through the portal vein, which is considerably less invasive than liver transplantation and hence not associated with surgery related complications. We identified 3 GSDIa and GSDIb patients, treated with hepatocyte transplantations (Additional file 1: Table S3, Additional file 1: Table S4). Normalization of metabolic parameters was observed in all patients after transplantation and no therapy-related complications were mentioned. This concurs with previously reported beneficial effects from human hepatocyte transplantations for different hepatic indications (metabolic, acute and chronic liver failure) [45]. However, these beneficial effects were short-lived and effects subsided within months. Similarly, metabolic improvement decreased in one of the GSDI patients treated with hepatocytes after 3 years and eventually subsided completely [46]. Nevertheless, these case reports show that cell-based therapies can restore liver function for at least a limited period, which might be beneficial in acute situations awaiting a liver transplantation.

Liver stem cell transplantations might provide additional advantages. Stem cells are highly proliferative and have the potential to bypass the current shortage of donor livers by expansion *in vitro* or *in vivo* upon engraftment. Furthermore, liver stem cell transplantations might require less immune suppression. For allograft survival after solid organ transplantation, all patients require life-long immune suppression, with serious associated side effects, including toxicity, malignancy development and infectious complications. Human fetal-liver derived hepatocytes have been given for end-stage decompensated liver cirrhosis without immune suppression, based on the concept that fetal cells do not express HLA yet [47]. Short term outcomes were promising, but long term follow up has not been reported. This illustrates that progenitor cell-based therapies might be given without or with reduced immune suppression. In this context, autologous transplantation with genetically corrected stem cells [48,49] and hepatocyte-like cells generated from autologous induced pluripotent stem cells [50] have exciting potential for the future.

Despite promising, some complications might still occur after stem cell based therapies, including renal complications. New-born *G6PC* knock-out mice treated with bone marrow-derived myelomonocytic cells displayed restored G6Pase activity and improved liver functional parameters, without amelioration of renal involvement [51]. Similarly, there is concern that hepatic adenoma and carcinoma might develop in the cells with a defect G6PT/G6Pase complex with the use of cell-based therapies that do not replace all patient cells. However, a recent study has demonstrated that the occurrence of hepatocellular adenoma was prevented by gene therapy in G6pc^-/-^ mice, despite partial and variable G6Pase activity, but with normalized blood metabolite profiles and glucose tolerance [52]. This concurs with the observation that development of hepatocellular adenoma and carcinoma appears to be related to the degree of steatosis and has been shown to regress with improved metabolic control [53], as is expected from cell-based therapies.

In conclusion, all GSDI patients reviewed in this article showed improved metabolic control and normal fasting tolerance after liver transplantation. This dramatically improved the quality of life of these patients, but a substantial number of patients experienced complications. Although some complications might be caused by disease progression, most seemed related to the liver transplantation procedure and subsequent immune suppression. These complications underscore the need for improvement of therapeutic strategies and emphasize the potential of novel (stem) cell-based treatments.

## Competing interests

The authors declare that they have no competing interests.

## Authors’ contributions

SB identified all the cases to be included, analyzed and interpreted the data and drafted the manuscript. PS reviewed the manuscript. PS, GV and SF contacted treating physicians and reviewed the manuscript. In addition, GV and SF analyzed and interpreted the data. All authors read and approved the final manuscript.

## Supplementary Material

Additional file 1 Information on individual GSDI patients undergoing liver transplantations. Click here for file
