# Supplementary material for: Liver transplantation in glycogen storage disease type I
Source: Orphanet J Rare Dis. 2014 Apr 9;9:47. doi: 10.1186/1750-1172-9-47 (PMC4113191; doi:10.1186/1750-1172-9-47)
Supplement: Additional file 1 — Information on individual GSDI patients undergoing liver transplantations. [file 1750-1172-9-47-S1.doc]

**Additional file 1: Table S1 Liver transplantation in GSD1a**

***NB: Each patient had normalization of metabolic parameters (no hypoglycemia, normal liver function) post-transplantation, except for one patient receiving hepatocyte transplantation for GSDIa at long term follow up. Therefore, improvement of metabolic control*** is not always mentioned in the table.

| **Ref** | **N** | **PT age (years) at transplantation** | **Year of transplantation** | **Diagnosis** | **Indication** | **Immunosuppressive regime** | **Follow-up (≤1 year post-transplantation)** | **Follow-up (>1 year post-transplantation)** | **Current age** |
| --- | --- | --- | --- | --- | --- | --- | --- | --- | --- |
| 1,2 | 1 | 16.5 | 1982 (2^nd^ OLT in 1996) |  | PMC, HA | Cyclosporine, steroid | *1y* normal liver function^13^ | *9y* portal vein thrombosis, portosystemic shunt^14^  *14y* 2^nd^ OLT with KT^23^  *15.3y* PT died at age 31^23^ | 48 |
| 3 | 1 | 23 | 1986 | Enzyme assay | HA | Cyclosporine and prednison | *0.5y* normal liver function |  | 51 |
| 4 | 1 | 11 | Between June 1985 and December 1986 | Enzyme assay | PMC, LA | Not reported | *3d* insulin-dependent diabetes (insulin pump for 5d)  *0.5y* normal liver function |  | 39 |
| 5 | 2 | 26.5 | Publication 1988, also KT | Enzyme assay | PMC, HA, RF | Not reported | *acute* moderate rejection  *1y* normal liver (and kidney) function | *11.3y* chronic rejection^23^ | 53 |
|  |  | 27 | Publication 1988 | Enzyme assay | PMC, LA | Not reported | *2d* insulin-dependent diabetes  *1y* normal liver (and kidney) function |  | 53 |
| 6 | 1 | 27 | 1987 | Enzyme assay | PMC. GR, HA | Corticosteroids and cyclosporine | *1.5m* normal liver function | *2y* normal liver function, catch-up growth (5.4cm) and increased testosterone levels | 54 |
| 7 | 1 | 6 | 1986 | Enzyme assay | PMC, GR | Prednisolone, azathioprine, cyclosporine A | *5d* arteria hepatica thrombosis  *9d* 2^nd^OLT with episode acute rejection | *2y* catch-up growth  *6y* good condition | 34 |
| 8 | 1 | 37 | Publication 1994 |  | HA | Not reported | Not reported |  | 57 |
| 9 | 2 (twins) | 19 | Publication 1996 |  | HA | Cyclosporine | *acute* renal failure, required dialysis |  | 37 |
|  |  | 19 | Publication 1996 |  | HA | Cyclosporine | *acute* renal failure, did not require dialysis |  | 37 |
| 10 | 1 | 5.8 | Between June 1994 and November 1996 (living related) |  |  | Cyclosporine, azathioprine, corticosteroids | *acute* rejection |  | 25 |
| 11 | 2 | 11.8 | 1993 |  | PMC, HA | Not reported | normal liver function and catch-up growth | *3.5y* rejection | 33 |
|  |  | 18.9 | 1991 |  | PMC, HA | Not reported |  | *6.8y* normal liver function | 42 |
| 12 | 3 | 15 | Publication 1999 | Enzyme assay | PMC, HA | Cyclosporine, prednisone | normalization liver function; catch-up growth | *1-8y* evolvement renal disease (focal glomerulosclerosis) with eventually dialysis  *8y* catch-up length growth (-6SD to -1.5SD) | 30 |
|  |  | 17 | Publication 1999 | Enzyme assay | HA, GR | Cyclosporine, prednisone | *acute* rejection (*d23*) | *4y* gouty arthritis  *5y* fluctuating levels ALAT, biopsy signs nonspecific hepatitis (no rejection)  *6y* catch-up length growth (-2.5SD to -1.5SD) and post-transplantation puberty completed | 32 |
|  |  | 23 | Publication 1999 | Enzyme assay | PMC, HA | Cyclosporine, prednisone | *acute* rejection (*d5* and *3m*); normalization liver function | *4y* hepatitis C  *8y* minor increase fibrosis and inflammation | 38 |
| 13 | 1 | 17 | Publication 2000 |  | PMC, HA, GR | Cyclosporine, mycophenolate |  | *2y* normal liver function | 31 |
| 14 | 1 | 16 | Publication 2002 |  | GR, PMC, pancreatitis | Not reported | *24h* 2^nd^ OLT (1^st^ OLT never functioned) | *2y* good condition (with unilateral optic atrophy) | 28 |
| 15 | 4 | 4.3-14.5 | Between 1996 and 2001 (living related) |  |  | Cyclosporine, corticosteroid, azathioprine | No details on individual patients  *acute* 2 PT acute cellular rejection  *1m* 1 PT prolonged drainage  *1m* 2 PT hypertension  *1m* 2 PT CMV | *2y* catch-up growth, normal liver function  *3y* stable function in 1PT with pre-transplantation renal involvement | 20-30 |
| 16 | 4 | 7 | Between February 1983 and March 2002 | Enzyme assay | PMC | Cyclosporine, azathioprine | Normal liver function  *acute* hepatic artery thrombosis, re-OLT with no complications |  | ? |
|  |  | 13 | Between February 1983 and March 2002 | Enzyme assay | HA | Tacrolimus, steroids | Normal liver function |  | ? |
|  |  | 14 | Between February 1983 and March 2002 | Enzyme assay | GR, HA | Tacrolimus, steroids | Normal liver function |  | ? |
|  |  | 23 | Between February 1983 and March 2002 | Enzyme assay | HA | Cyclosporine, azathioprine | Normal liver function | *6y* late portal vein thrombosis, chronic non-specific hepatitis | ? |
| 17 | 1 | 34 | 1996 (also KT) |  | RF | Not reported |  | *4.1y* alive | 52 |
| 18 | 1 | 25 | Publication 2004 (also KT) |  | HA, RF | Cyclosporine A, steroids | *4m* normal liver and kidney function |  | 35 |
| 19 | 1 | 6 | Publication 2005 |  | HA | Tacrolimus, corticosteroids |  | *4y* good condition | 15 |
| 20 | 9 | median 7.3 | Between June 1994 and December 2005 (living related) | Enzyme and DNA analysis | PMC | Cyclosporine, prednisolone,  and azathioprine | No details on individual patients, reported complications include also complications of 3 GSD3 patients reported  *acute* no recurrent hypoglycemia, all patients catch-up growth, sexual maturation unpredictable  *1m* 1 PT prolonged drainage ascetic fluid  *1m* 1 PT minor bile leak  *1m* 2 PT hepatic venous outflow obstruction (balloon dilation)  *1m* 2 PT early postoperative hypertension (but no long on antihypertensive)  *1m* 2 PT CMV  *2m* 1 PT post transplantation severe acute pancreatitis and sepsis (has died)  1 PT with GSD1a with nephrocalcinosis  1 PT with GSD1a delayed menarche  2 PT with GSD1a mild mesangial glomerulonephritis |  | ? |
| 21 | 1 | 19.5 | 2000 (also KT) | DNA analysis | PMC, RF | Cyclosporine A,  mycophenolate mofetil, prednisone |  | *2y* normal liver and kidney function | 34 |
| 22 | 1 | 26 | Publication 2007 |  | PMC, HA, focal nodular hyperplasia | Not reported |  | good long-term outcome | 34 |
| 23 | 1 | 17.3 | Publication 2007 |  | HA | Not reported | *acute* rejection | *3.4y* renal dysfunction | 24 |
| 24 | 1 | 5 | Publication 2008 (living related) | DNA analysis | PMC, GR, anemia | Not reported | *28d* normal liver function, spontaneously stopping nasal bleeds |  | 11 |
| 25 | 5 | 26 | Between 2004 and 2006 |  | PMC | Tacrolimus, mycofenolate mofetil | *acute* CMV infection | *25-48m* normal liver function | 35 |
|  |  | 29 | Between 2004 and 2006 |  | HA | Tacrolimus | *acute* rejection | *25-48m* normal liver function | 38 |
|  |  | 30 | Between 2004 and 2006 |  | HA | Tacrolimus | *acute* progressive renal dysfunction | *25-48m* normal liver function | 39 |
|  |  | 31 | Between 2004 and 2006 |  | HA | Tacrolimus | *acute* progressive renal dysfunction, rejection, CMV infection | *25-48m* normal liver function | 40 |
|  |  | 38 | Between 2004 and 2006 |  | HA | Sirolimus, cyclosporine | *acute* progressive renal dysfunction, diabetes mellitus | *25-48m* normal liver function, without nephrolithiasis; chronic renal insufficiency | 47 |
| 26 | 1 | 30 | Publication 2011 (also KT) | DNA analysis | PMC, HA, RF | Steroids,  tacrolimus, mycophenolate mofetil | *5d* rapid normalization metabolic parameters  *1m* CMV infection  *7m* good condition, both grafts functional |  | 33 |
| 27 | 1 | 27 | Publication 2013 | Enzyme analysis | HA | Not reported |  | *2y* during pregnancy moderate rejection | 28 |
| **Personal communication (P Labrune)** | 1 | 20 | 1993 |  |  |  |  | *3y* renal failure with dialysis, patient not alive (suicide) | 41 |
| **Personal communication (P Labrune)** | 1 | 16 | 1998 |  | Mutation analysispenia |  |  |  | 32 |
| **Personal communication (DA Weinstein)** | 1 | 34 | 2001 |  | Mutation analysis |  | *4m* metastatic heptocellular carcinoma |  | 47 |
| **Personal communication (DA Weinstein)** | 1 | 17 | 2003 |  | Mutation analysis |  |  | *2y* neuropathy and chronic tremor | 28 |
| **Personal communication (DA Weinstein)** | 1 | 22 | 2007 |  | Mutation analysis |  |  |  | 29 |
| **Personal communication (DA Weinstein)** | 1 | 50 | 2007 |  | Mutation analysis |  |  |  | 57 |
| **Personal communication (P Labrune)** | 1 | 39 | 2011 |  | Mutation analysis |  |  | *2y* no recurrence hepatocellular carcinoma | 44 |
| **Personal communication (P Labrune)** | 1 | 28 | 2012 |  | Mutation analysis |  |  |  | 30 |

**Additional file 1: Table S2 Liver transplantation in GS**D1b

| **Ref** | **N** | **PT age (y)** | **Year of transplantation** | **Diagnosis** | | **Indication** | **Immunosuppressive regime** | **Follow-up (≤1 year post-transplantation)** | | **Follow-up (>1 year post-transplantation)** | **Current age** |
| --- | --- | --- | --- | --- | --- | --- | --- | --- | --- | --- | --- |
| 31,32 | 1 | 7.5 | 1991 |  | | PMC, recurrent infections | Prednisone,  cyclosporine (first month also azathioprine) | *acute* initial normal values neutrophil plus platelet counts and reduction infections, later persistent neutropenia | | *2y* normal metabolic control, prolonged bleeding time and frequent bruises  *6.2y* CMV infection^23^ | 31 |
| 33 | 1 | 7 | Publication 1994 |  | | PMC | Tacrolimus, methylprednisone |  | | *2y* no improvement neutropenia | 27 |
| 11 | 1 | 13.8 | 1993 |  | | PMC | Not reported | *acute* neutropenia | | *4.4y* persistent neutropenia and rejection | 35 |
| 34 | 1 | 32 | February 1997 |  | | PMC, ulcera | Tacrolimus, methylprednisolone, azathioprine | *7m* hepatitis B infection | | *4y* persistent correction metabolic parameters, cyclic neutropenia | 49 |
| 35 | 1 | 18 | Publication 2003 (living related) |  | | PMC | Tacrolimus, steroids | *38d* normal liver function | | *4y* slight improvement neutrophil number (discontinue G-CSF) | 29 |
| 36 | 2 | 8 | Publication 2004 |  | | GR, PMC | Not reported | *acute* catch-up growth, reduced infections, persistent neutropenia | | *4y* persistent neutropenia | 18 |
|  |  | 11.1 | Publication 2004 |  | | GR, PMC | Not reported | *acute* catch-up growth, reduced infections, persistent neutropenia | |  | 21 |
| 37 | 1 | 13.1 | Between June 1990 and December 2003 (living related) |  | |  | Not reported | *1.4m* systemic candidiasis and death | |  | ? |
| 38 | 1 | 32 | 2005 (2y before OLT KT) | Enzyme assay | | PMC, GR, recurrent infections | Tacrolimus, prednisolone | *8m* normal liver function, anaemia and neutropenia | | *3.5y* normal kidney function | 41 |
| 39 | 4 | 1 | Between November 2005 and June 2008 (living related) | DNA analysis | | PMC | Tacrolimus, steroids | *acute* stabilization glucose, normalized neutrophil count | | *1-3.5y* improved neutropenia | 9 |
|  |  | 3.5 | Between November 2005 and June 2008 (living related) | DNA analysis | | PMC | Tacrolimus, steroids | *acute* stabilization glucose, normalized neutrophil count | | *1-3.5y* improved neutropenia | 10 |
|  |  | 4 | Between November 2005 and June 2008 (living related) | DNA analysis | | PMC | Tacrolimus, steroids | *acute* stabilization glucose, normalized neutrophil count, tacrolimus encephalopathy | | *1-3.5y* improved neutropenia | 10 |
|  |  | 8.3 | Between November 2005 and June 2008 (living related) | DNA analysis | | PMC | Tacrolimus, steroids | *acute* stabilization glucose, normalized neutrophil count | | *1-3.5y* improved neutropenia | 14 |
| 40 | 8 | 1 1.5  1.5  2.5  3.5  4  6.3  13 | Between November 2005 and September 2011 | DNA analysis | | PMC, recurrent infection | Tacrolimus, steroids | No details on individual patients  *acute* 2 PT required G-CSF due to combined IBD (all others withdrawn from G-CSF), 6 PT infections complications (2 PT catheter-related infection, 2 PT urinary duct infection, 1 PT pneumonia, 1 PT infectious ascites) | | *1m-5.3y* during follow-up all alive | ? |
| 41 | 1 | 2.9-14.5 | Publication 2013 (living related) | |  |  | Not reported |  |  | | 4-16 |
| **Personal communication (DA Weinstein)** | 1 | 44 | 2012 | Mild neutropenia, malignancy | | Mutation analysis |  |  | | Still malignancy | 46 |

**Additional file 1: Table S3** Hepatocyte transplantation in GSD1a

| **Ref** | **N** | **PT age (y)** | **Year of transplantation** | **Diagnosis** | **Indication** | **Immunosuppressive regime** | **Follow-up (≤1 year post-transplantation)** | **Follow-up (>1 year post-transplantation)** | **Current age** |
| --- | --- | --- | --- | --- | --- | --- | --- | --- | --- |
| 28 | 1 | 47 | Publication 2002 | Enzyme assay | PMC, HA | Methylpredinosolone, tacrolimus, mycophenolate mofetil | *8w* G6PC enzyme activity measured in liver biopsy  *9m* normal liver function | *3y* metabolic improvement reduced, finally abolished (due to rejection or to senescence of transplanted cells) ^29^ | 59 |
| 30 | 1 | 6 | Publication 2012 | DNA analysis | PMC | Methylprednisolone, tacrolimus |  | *1y* normal liver function | 8 |

Supplementary Table 4 Hepatocyte transplantation in GSD1b

| **Ref** | **N** | **PT age (y)** | **Year of transplantation** | **Diagnosis** | **Indication** | **Immunosuppressive regime** | **Follow-up (≤1 year post-transplantation)** | **Current age** |
| --- | --- | --- | --- | --- | --- | --- | --- | --- |
| 42 | 1 | 18 | Publication 2007 | Enzyme assay | PCM | Not reported | *7m* good initial response, fasting tolerance improved, clinically well | 25 |

*d: day, m: month, y: year; GR: growth retardation; HA: hepatic adenomas; LA: liver abnormalities (in most cases it concerns focal nodular hyperplasia); KT: kidney transplantation; OLT: orthotopic liver transplantation; PMC: poor metabolic control; PT: patient; RF: renal failure*

1. Malatack JJ, Finegold DN, Iwatsuki S, et al. Liver transplantation for type I glycogen storage disease. *Lancet*. 1983;1(8333):1073-1075.

2. Selby R, Starzl TE, Yunis E, et al. Liver transplantation for type I and type IV glycogen storage disease. *Eur J Pediatr*. 1993;152 Suppl 1:S71-6.

3. Coire CI, Qizilbash AH, Castelli MF. Hepatic adenomata in type ia glycogen storage disease. *Arch Pathol Lab Med*. 1987;111(2):166-169.

4. Martinez Ibanez V, Margarit C, Tormo R, et al. Liver transplantation in metabolic diseases. report of five pediatric cases. *Transplant Proc*. 1987;19(5):3803-3804.

5. Poe R, Snover DC. Adenomas in glycogen storage disease type 1. two cases with unusual histologic features. *Am J Surg Pathol*. 1988;12(6):477-483.

6. Kirschner BS, Baker AL, Thorp FK. Growth in adulthood after liver transplantation for glycogen storage disease type I. *Gastroenterology*. 1991;101(1):238-241.

7. Sokal EM, Lopez-Silvarrey A, Buts JP, Otte JB. Orthotopic liver transplantation for type I glycogenosis unresponsive to medical therapy. *J Pediatr Gastroenterol Nutr*. 1993;16(4):465-467.

8. Kay RM, Eckardt JJ, Goldstein LI, Busuttil RW. Metastatic hepatocellular carcinoma to bone in a liver transplant patient. A case report. *Clin Orthop Relat Res*. 1994;(303)(303):237-241.

9. Reid CJ, Hebert D. Acute renal failure complicating liver transplantation in twin sisters with glycogen storage disease type ia. *Transplant Proc*. 1996;28(6):3629-3631.

10. Chen CL, Chen YS, Liu PP, et al. Living related donor liver transplantation. *J Gastroenterol Hepatol*. 1997;12(9-10):S342-5.

11. Matern D, Starzl TE, Arnaout W, et al. Liver transplantation for glycogen storage disease types I, III, and IV. *Eur J Pediatr*. 1999;158 Suppl 2:S43-8.

12. Faivre L, Houssin D, Valayer J, Brouard J, Hadchouel M, Bernard O. Long-term outcome of liver transplantation in patients with glycogen storage disease type ia. *J Inherit Metab Dis*. 1999;22(6):723-732.

13. Koestinger A, Gillet M, Chiolero R, Mosimann F, Tappy L. Effect of liver transplantation on hepatic glucose metabolism in a patient with type I glycogen storage disease. *Transplantation*. 2000;69(10):2205-2207.

14. Labrune P. Glycogen storage disease type I: Indications for liver and/or kidney transplantation. *Eur J Pediatr*. 2002;161 Suppl 1:S53-5. doi: 10.1007/s00431-002-1004-y.

15. Liu PP, de Villa VH, Chen YS, et al. Outcome of living donor liver transplantation for glycogen storage disease. *Transplant Proc*. 2003;35(1):366-368.

16. Lerut JP, Ciccarelli O, Sempoux C, et al. Glycogenosis storage type I diseases and evolutive adenomatosis: An indication for liver transplantation. *Transpl Int*. 2003;16(12):879-884. doi: 10.1007/s00147-003-0613-3.

17. Demirci G, Becker T, Nyibata M, et al. Results of combined and sequential liver-kidney transplantation. *Liver Transpl*. 2003;9(10):1067-1078. doi: 10.1053/jlts.2003.50210.

18. Panaro F, Andorno E, Basile G, et al. Simultaneous liver-kidney transplantation for glycogen storage disease type IA (von gierke's disease). *Transplant Proc*. 2004;36(5):1483-1484. doi: 10.1016/j.transproceed.2004.05.070.

19. Arikan C, Kilic M, Nart D, et al. Hepatocellular carcinoma in children and effect of living-donor liver transplantation on outcome. *Pediatr Transplant*. 2006;10(1):42-47. doi: 10.1111/j.1399-3046.2005.00395.x.

20. Iyer SG, Chen CL, Wang CC, et al. Long-term results of living donor liver transplantation for glycogen storage disorders in children. *Liver Transpl*. 2007;13(6):848-852. doi: 10.1002/lt.21151.

21. Belingheri M, Ghio L, Sala A, et al. Combined liver-kidney transplantation in glycogen storage disease ia: A case beyond the guidelines. *Liver Transpl*. 2007;13(5):762-764. doi: 10.1002/lt.21147.

22. Carreiro G, Villela-Nogueira CA, Coelho H, et al. Orthotopic liver transplantation in glucose-6-phosphatase deficiency--von gierke disease--with multiple hepatic adenomas and concomitant focal nodular hyperplasia. *J Pediatr Endocrinol Metab*. 2007;20(4):545-549.

23. Davis MK, Weinstein DA. Liver transplantation in children with glycogen storage disease: Controversies and evaluation of the risk/benefit of this procedure. *Pediatr Transplant*. 2008;12(2):137-145. doi: 10.1111/j.1399-3046.2007.00803.x; 10.1111/j.1399-3046.2007.00803.x.

24. Kaihara S, Ushigome H, Sakai K, et al. Preemptive living donor liver transplantation in glycogen storage disease ia: Case report. *Transplant Proc*. 2008;40(8):2815-2817. doi: 10.1016/j.transproceed.2008.07.026; 10.1016/j.transproceed.2008.07.026.

25. Reddy SK, Austin SL, Spencer-Manzon M, et al. Liver transplantation for glycogen storage disease type ia. *J Hepatol*. 2009;51(3):483-490. doi: 10.1016/j.jhep.2009.05.026; 10.1016/j.jhep.2009.05.026.

26. Marega A, Fregonese C, Tulissi P, et al. Preemptive liver-kidney transplantation in von gierke disease: A case report. *Transplant Proc*. 2011;43(4):1196-1197. doi: 10.1016/j.transproceed.2011.03.003; 10.1016/j.transproceed.2011.03.003.

27. Carvalho PM, Silva NJ, Dias PG, Porto JF, Santos LC, Costa JM. Glycogen storage disease type 1a - a secondary cause for hyperlipidemia: Report of five cases. *J Diabetes Metab Disord*. 2013;12(1):25-6581-12-25. doi: 10.1186/2251-6581-12-25; 10.1186/2251-6581-12-25.

28. Muraca M, Gerunda G, Neri D, et al. Hepatocyte transplantation as a treatment for glycogen storage disease type 1a. *Lancet*. 2002;359(9303):317-318. doi: 10.1016/S0140-6736(02)07529-3.

29. Muraca M, Burlina AB. Liver and liver cell transplantation for glycogen storage disease type IA. *Acta Gastroenterol Belg*. 2005;68(4):469-472.

30. Ribes-Koninckx C, Ibars EP, Calzado Agrasot MA, et al. Clinical outcome of hepatocyte transplantation in four pediatric patients with inherited metabolic diseases. *Cell Transplant*. 2012;21(10):2267-2282. doi: 10.3727/096368912X637505; 10.3727/096368912X637505.

31. Lachaux A, Boillot O, Stamm D, et al. Treatment with lenograstim (glycosylated recombinant human granulocyte colony-stimulating factor) and orthotopic liver transplantation for glycogen storage disease type ib. *J Pediatr*. 1993;123(6):1005-1008.

32. Donadieu J, Bader-Meunier B, Bertrand Y, et al. Recombinant human G-CSF (lenograstim) for infectious complications in glycogen storage disease type ib. report of 7 cases. *Nouv Rev Fr Hematol*. 1994;35(6):529-534.

33. Tanaka A, Tanaka K, Kitai T, et al. Living related liver transplantation across ABO blood groups. *Transplantation*. 1994;58(5):548-553.

34. Martinez-Olmos MA, Lopez-Sanroman A, Martin-Vaquero P, et al. Liver transplantation for type ib glycogenosis with reversal of cyclic neutropenia. *Clin Nutr*. 2001;20(4):375-377. doi: 10.1054/clnu.2001.0432.

35. Adachi M, Shinkai M, Ohhama Y, et al. Improved neutrophil function in a glycogen storage disease type 1b patient after liver transplantation. *Eur J Pediatr*. 2004;163(4-5):202-206. doi: 10.1007/s00431-004-1405-1.

36. Bhattacharya N, Heaton N, Rela M, Walter JH, Lee PJ. The benefits of liver transplantation in glycogenosis type ib. *J Inherit Metab Dis*. 2004;27(4):539-540.

37. Morioka D, Kasahara M, Takada Y, et al. Living donor liver transplantation for pediatric patients with inheritable metabolic disorders. *Am J Transplant*. 2005;5(11):2754-2763. doi: 10.1111/j.1600-6143.2005.01084.x.

38. Martin AP, Bartels M, Schreiber S, Buehrdel P, Hauss J, Fangmann J. Successful staged kidney and liver transplantation for glycogen storage disease type ib: A case report. *Transplant Proc*. 2006;38(10):3615-3619. doi: 10.1016/j.transproceed.2006.10.160.

39. Kasahara M, Horikawa R, Sakamoto S, et al. Living donor liver transplantation for glycogen storage disease type ib. *Liver Transpl*. 2009;15(12):1867-1871. doi: 10.1002/lt.21929; 10.1002/lt.21929.

40. Karaki C, Kasahara M, Sakamoto S, et al. Glycemic management in living donor liver transplantation for patients with glycogen storage disease type 1b. *Pediatr Transplant*. 2012;16(5):465-470. doi: 10.1111/j.1399-3046.2012.01705.x; 10.1111/j.1399-3046.2012.01705.x.

41. Hussein MH, Hashimoto T, Suzuki T, et al. Children undergoing liver transplantation for treatment of inherited metabolic diseases are prone to higher oxidative stress, complement activity and transforming growth factor-beta1. *Ann Transplant*. 2013;18:63-68. doi: 10.12659/AOT.883820; 10.12659/AOT.883820.

42. Lee KW, Lee JH, Shin SW, et al. Hepatocyte transplantation for glycogen storage disease type ib. *Cell Transplant*. 2007;16(6):629-637.
